# A multi-domain trust management model for supporting RFID applications of IoT

**DOI:** 10.1371/journal.pone.0181124

**Published:** 2017-07-14

**Authors:** Xu Wu, Feng Li

**Affiliations:** 1 School of Computer, Electronics and Information, Guangxi University, Nanning, China; 2 School of Computer Science, Xi’an University of Posts and Telecommunications, Xi’an, China; 3 School of Engineering and Technology, Indiana University–Purdue University Indianapolis, Indianapolis, United States of America; University of Texas at San Antonio, UNITED STATES

## Abstract

The use of RFID technology in complex and distributed environments often leads to a multi-domain RFID system, in which trust establishment among entities from heterogeneous domains without past interaction or prior agreed policy, is a challenge. The current trust management mechanisms in the literature do not meet the specific requirements in multi-domain RFID systems. Therefore, this paper analyzes the special challenges on trust management in multi-domain RFID systems, and identifies the implications and the requirements of the challenges on the solutions to the trust management of multi-domain RFID systems. A multi-domain trust management model is proposed, which provides a hierarchical trust management framework include a diversity of trust evaluation and establishment approaches. The simulation results and analysis show that the proposed method has excellent ability to deal with the trust relationships, better security, and higher accuracy rate.

## Introduction

The term Internet of Things (IoT) arises from the need to establish heterogeneous environments where the devices with varying processing capabilities can cooperate and communicate in an intelligent environment transparently to the user [1]. In its background and current research of IoT section, Radio Frequency Identification (RFID) technology is considered as a foundational technology for IoT. RFID has been widely used in many and diverse areas, such as logistics, pharmaceutical production, retailing and supply chain management [2]. The use of RFID technology in complex and distributed environments often leads to a multi-domain RFID system in which security issues such as authentication of tags and readers, granting access to data, and revocation of readers turn into an administrative challenge. A common scenario is eEnabled airplanes scenario [3], where on-board RFID tags and readers will be connected to different ground systems across multiple management domains, for logistics and access control. The part maintenance history contained in on-board RFID tags is the airline’s proprietary information and the access should be protected against random or intentional access from illegal RFID readers of other management domains.

Many cryptographic authentication and data protection techniques have been proposed to solve the security issues in the literature [4–8]. Although conventional cryptographic mechanisms can provide data confidentiality, data integrity and node authentication for exchanged messages and protect the system from external attacks, they fail to deal with insider attackers [9]. For example a reader owning legitimate cryptographic keys can easily launch an internal attack inside the system by altering data or injecting bogus information without being identified. So we need to introduce trust management into IoT RFID system.

Trust management is a mechanism that also allows identifying malicious, selfish, and compromised nodes. Trust computation model and trust management systems have been implemented successfully in commercial applications. There is also a rapidly growing literature around topics of trust and reputation management for IoT [10, 11]. Devices in the IoT may be equipped with inexpensive low-performance microcontrollers that provide just enough computing power to periodically perform their intended tasks, i.e. obtain sensor readings and communicate with other nodes. The problem of trustworthiness and trust management of low-power low performance computing nodes has been discussed in previous research, in particular in the context of Wireless Sensor Networks (WSNs) [12]. Importantly, most techniques proposed in this field focus on building trust relationship between nodes of the same domain based on observing the communication behavior of these nodes. The current trust management mechanisms in the literature do not meet all requirements for a functional implementation for the IoT context.

In the multi-domain RFID paradigm, a mobile tag will potentially interact with numerous readers from different management domains for a coalition, as well as leverage available (foreign) infrastructure for information access while on the move. However, trust establishment among entities from heterogeneous domains without past interaction or prior agreed policy, is a challenge. We analyze the special challenges on trust management in the multi-domain RFID system, when compared to conventional RFID system in IoT environments, and identify the implications and the requirements of the challenges on the solutions to the trust management of multi-domain RFID systems.

**Heterogeneity of management domains:** Two different management domains, who want to establish a coalition, may carry their own policies for authentication and authorization. They need to negotiate for permitting access to each other’s RFID tags. The trust management of multi-domain RFID systems is required to provide a flexible and configurable trust model, enable readers and authentication centers of different domains to negotiate and collaborate.

**Diverse trust requirements:** There exists multi-type entities include RFID tags, RFID readers and authentication centers in the multi-domain RFID systems. These entities have diverse trust requirements due to the different of their number, capability and stability. The trust management of multi-domain RFID systems has to be providing a diversity of trust evaluation approaches to accommodate potentially a diversity of trust requirements.

**Support of multiple applications:** There is a wealth of potential RFID applications such as object identification, any subsequent tracking and record management. Each application has its unique requirements on implementation. However, a generic trust module underlying all the RFID application will be ideal as it increases reusability & scalability. A trust management solution is preferred to be adaptive to the diverse applications.

**Large scale Systems:** With the advances in IoT technologies, the number of nodes available in multi-domain RFID systems will be enormous. Thus, the trust management solution needs to be scalable. The trust management approaches are required to include efficient algorithms in terms of computation, communication and/or storage for trust evaluation and establishment, so to handle access requests and information exchange from a potentially large number of collaborative entities.

Based on the specific requirements in multi-domain RFID systems, this paper focuses on the critical trust management issues and proposes a multi-domain trust management model. The proposed trust management model provides a hierarchical trust management framework. The main contributions of our system are:

1. A hierarchical trust model including RFID reader trust layer and authentication center trust layer is proposed by us, which provides a diversity of trust evaluation and establishment approaches to accommodate heterogeneous management domains and diverse trust requirements.
2. The D-S theory is introduced to compute the trustworthiness of readers. To make the D-S theory fit into multi-domain RFID systems; we creatively define three interaction events and nine event assumptions, which is adaptive to the multiple applications.
3. Another trust evaluation method of reader is proposed based on verification of interaction proof. The proposed method verifies the authorization use of a reader by saving its interaction proof in the tag. Only saving the recent interaction feedback record in the tag is suitable for limited built-in memory tag.
4. A centralized trust evaluation scheme is proposed to evaluate the trustworthiness of authentication centers. An administration center is in charge of managing the trust of authentication center based on the abnormal event reports of readers of its own domain. Using more abnormal event reports helps trust convergence more quickly. Therefore our scheme can deal with large scale RFID applications.

This paper is organized as follows. Section 2 describes related work. In Section 3, the proposed trust management method is discussed. Section 4 describes the test scenario and simulation results. Finally, we conclude with a summary of our results and directions for new research in Section 5.

## Related work

### Trust management in IoT environments

In the literature, there is a rapidly growing literature around topics of trust and reputation management for IoT [11]. Several trust management systems have been proposed for RFID systems in IoT environments. Basically, trust management is the mechanisms to evaluate, establish, maintain, and revoke the trust between devices of the same or different networks within the IoT environment. The trust computation techniques in [13] are classified on four design dimensions: trust composition, trust propagation, trust aggregation and trust update. The authors summarize advantages and drawbacks of each dimension's options, and highlight the effectiveness of defense mechanisms against malicious attacks.

The work in [14] proposes an IoT protocol framework for RFID-based devices—the Scalable RFID Security Framework and Protocol Supporting IoT (SRSFPSI). The proposal entails an effective ID procedure founded on a hybrid framework (group-based and collaborative technique) and highly adaptive security monitoring handoff for RFID IoT networks. The protocol offers adaptability and scalability while upholding secure and adaptable RFID net-works. Other than preventing the introduction of malicious nodes and facilitating scalability, the protocol is integrated with a malware recognition tool.

In [15], the authors propose a lightweight and robust trust establishment scheme. The proposed trust scheme is lightweight thanks to a simple trust estimation method. The comprehensiveness and flexibility of the proposed trust estimation scheme make it robust against different types of attack and misbehavior. But evaluation results show one drawback of the proposed scheme is that it is sensitive to false-positive alarms, compared to other trust mechanisms.

The work in [16] presents a trust management scheme based on revised Dempster-Shafer (D-S) evidence theory. D-S theory is preponderant in tackling both random and subjective uncertainty in the trust mechanism. A trust propagation mechanism including conditional trust transitivity and dynamic recommendation aggregation is developed for obtaining the recommended trust values from third part nodes. Our proposed scheme is inspired by [16], but we use the different Dempster rules in our mole. In addition, the shortcomings of D-S evidence theory based trust scheme are analyzed in our paper.

The work in [17] proposes a computational model for the trust management. In order to enhance the security of data sharing and access control, the trust evaluation is built into the process of transactions of the data exchange and authorization. An example shows the performance of the proposed computational trust model. In [18], the authors investigate the personalized applications and services of IoT by detecting people-object gestures with a passive RFID tag. The proposal is analyzed based on people-object gestures classification. In [19], the authors also present a hierarchical trust model for the Internet of Things, similar to our work. Though the simulation results show the benefit of hierarchical trust model, the proposed model doesn’t explain the details about how to calculate the trust of reader. Our work is different with [19]. The trust relationship is classed into three classes: intra-domain trust, inter-domain trust and cross-domain, and time window mechanism is introduced in our multi-domain trust management model.

In [20], the authors evaluate the existing approaches to trust management in the Internet of Things based on three parameters. The first parameter focuses on trust management protocol in IoT, the second parameter concerns scalable solutions for trust management in IoT, and the third parameter addresses context-aware assessment in IoT. The paper has given a comparative evaluation of each existing approach for trust modeling in IoT, based on these parameters. Finally, the authors consider that the further research into trust management in IoT is required to develop scalable and context-aware trust solutions in IoT networks.

All these trust management schemes do not focus on the trust issue of multi-domain RFID systems. Designing a suitable trust management model to evaluate the trust of entities from heterogeneous domains without past interaction or prior agreed policy, is a challenge. In the paper, we analyze the special challenges on trust management in multi-domain RFID systems, and identify four trust requirements for multi-domain RFID systems. Finally, a hierarchical trust management framework is proposed to build the trust relationships among entities from heterogeneous domains.

### D-S evidence theory

In 1976, Shafer published a book named A Mathematical Theory of Evidence [21]. Dempster-Shafer Theory has a wide range of application on uncertainty reasoning, decision analysis and predication. Evidence theory is based on belief function and plausible reasoning [22].

First of all, we define Θ as a frame of discernment {*T*, *¬T*} as the set of propositions under consideration where *T* and *¬T* mean that the given agent considers a given correspondent to be trustworthy or not to be trustworthy, respectively. The sign 2^Θ^ indicates the set composed of all the subset generated by the frame of discernment. For a hypothesis set, denoted by A, m(A)→[0,1]

$$\begin{array}{l} m(Ø)=0\\ \sum_{A\in 2^{\Theta }}m(A)=1 \end{array}$$

Ø is the sign of an empty set. The function m is the basic belief assignment.

Dempster's rule of combination combines two independent evidences.

$$\begin{array}{l} m(Ø)=0\\ \left\{ \begin{array}{l} m(A)=\frac{1}{1-K}\sum_{B\cap C=A}m_{1}(B)m_{2}(C)\\ K=\sum_{B\cap C=Ø}m_{1}(B)m_{2}(C) \end{array}\right. \end{array}$$

Dempster's rule of more than two evidences: Suppose there are *m* evidences that are independent.

$$\left\{ \begin{array}{l} \{m(A)=(m_{1}(A_{1})⊕m_{2}(A_{2})⊕\ldots )⊕m_{p}(A_{p})\\ m(\phi )=0 \end{array}\right.$$

The basic probability assignments are *m*_1_,*m*_2_,….*m*_*p*_. The focal elements are *A*_1_,*A*_2_,….,*A*_*p*_. *m*(*A*) is a basic probability assignment which describes the combined evidence.

The trust evaluation strategy of readers in section 3.2 is proposed based on the D-S evidence theory in our paper.

## Proposed trust management model

Our work will focus on the authentication and a measure of trust between RFID tags and readers by using a hierarchical trust model, which regulates the authentication process based on the trustworthiness of entities. In the section, we express the details of the proposed trust management model.

### System model

**Fig 1:**
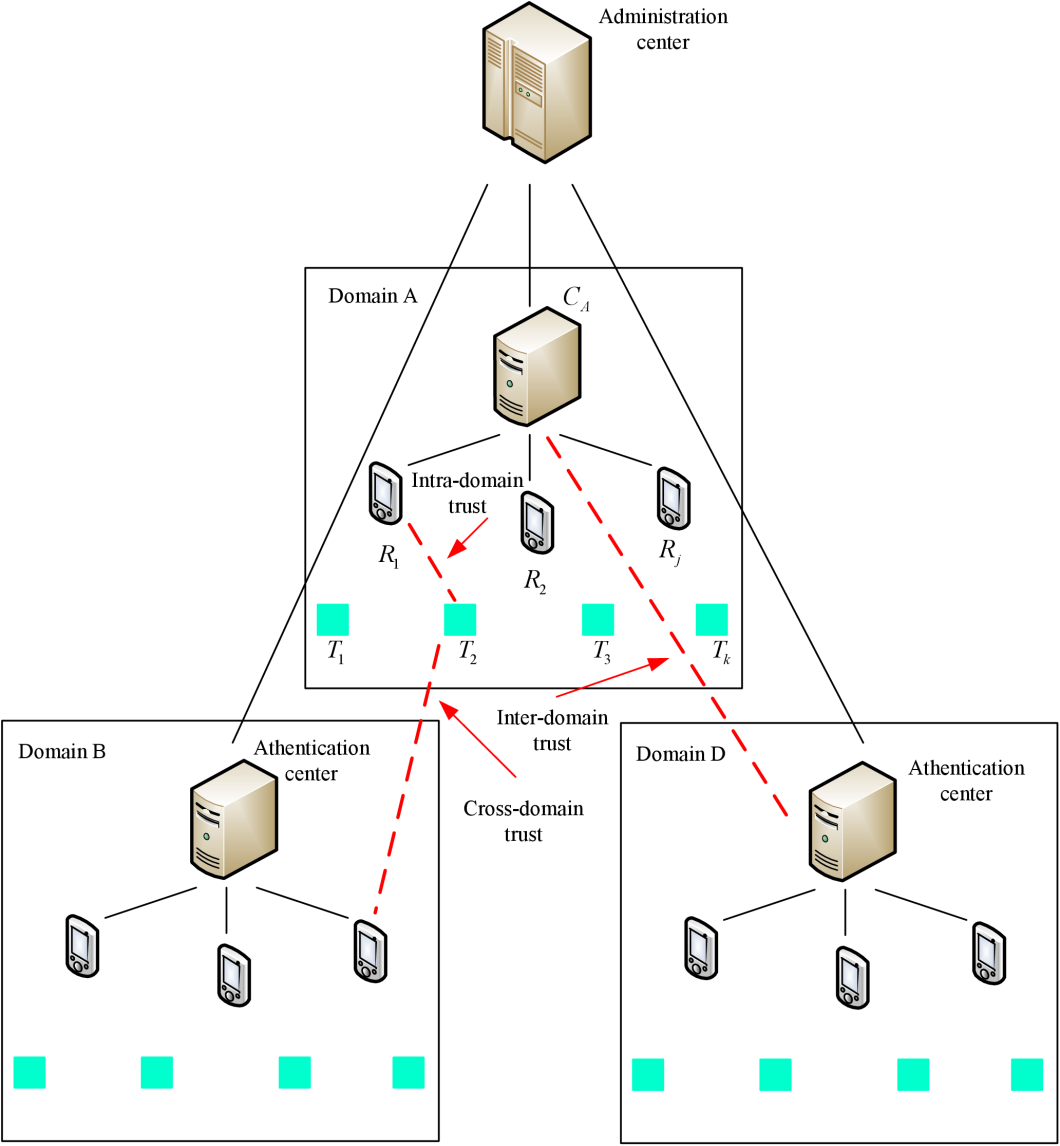
Our RFID system model.

Our RFID system model consists of one or more domains which in turn include four types of entities: RFID tags, RFID readers, authentication centers and an administration center (see Fig 1). In addition, RFID readers are also named as nodes. It is similar with the model in [23]. The RFID tag located on the object to be identified is the data carrier in the RFID system. The RFID reader is be able to interact with a tag include both reading data from and writing data to a tag. Every domain has an authentication center. The authentication center authorizes a reader of its own domain or other domain to interact with a tag of its own domain, and utilizes the data obtained from the tag in some useful manner. An administration center manages and maintains the trust of authentication centers.

In particular, a tag *T*_*k*_ and a reader *R*_*j*_ belong to an administrative domain A which is controlled by an authentication center *C*_*A*_-which in the following is referred to as home domain. While a tag is typically attached to an object that may roam to other administrative domains, also referred to as visited domains, a reader will always remain in its home domain only. Furthermore, we assume that a reader is always connected to its home authentication center via a secure channel. Also, an authentication center is always connected to the administration center via a secure channel, while the communication between tags and readers is insecure.

In the paper, we class the trust relationship in a multi-domain RFID system into three categories (marked with red color in Fig 1) based on trust domain boundaries: 1) Intra-domain trust refers to the trust relationship between tags and the readers of the domain. 2) Inter-domain trust is a kind of trust relationship which is set up by the authentication centers in the system levels. 3) Cross-domain trust means the trust relationship between tags and the readers of different domains.

**Fig 2:**
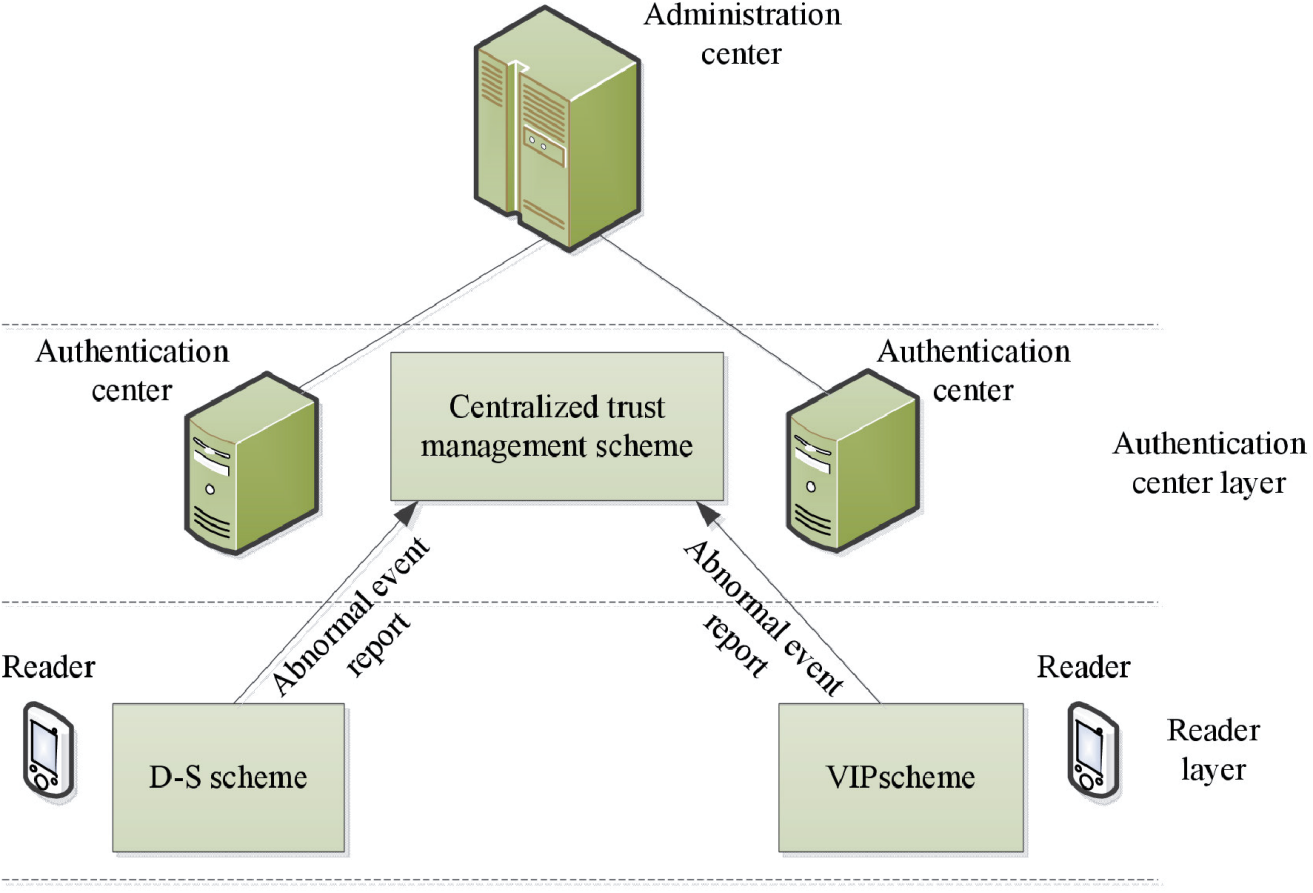
Hierarchical trust management framework.

A hierarchical trust management framework shown in Fig 2 is proposed to build the trust relationships among entities from heterogeneous domains. We assume that RFID tag is protected and trusted. Thus, we only focus on evaluating the trustworthiness of RFID reader and authentication center. We refer to two layers of trust in the framework: RFID reader trust layer and authentication center trust layer.

**In RFID reader trust layer**: We propose two kinds of scheme to evaluate the trust of readers: D-S evidence theory based scheme (D-S scheme) and verification of interaction proof based scheme (VIP scheme). Section 3.2 and 3.3 represent the details of evaluating the trustworthiness of RFID reader.

**In authentication center trust layer**: An administration center is used to manage the trustworthiness of authentication centers in a centralized way. The trust of an authentication center is eventually obtained by aggregating the abnormal event reports of all readers of its own domain. The system model section describes how to management and evaluate the trust of authentication center.

### Trust evaluation of RFID readers based on D-S evidence theory

In our trust model, the formation of an opinion about trustworthiness of a RFID reader depends on its interaction behaviors with other entities. Every node is implemented a watchdog agent that detects the interaction behaviors of neighbor nodes [24]. Table 1 shows three kinds of interaction events observed by neighbor nodes.

**Table 1:** Different interaction behaviors of a reader.

Event type	Assumptions of interaction behavior	Behavior type	mark
Discarding data (orders)	Not discarding data (orders)	normal	*A*_*0*_
	discarding data (orders) due to not connect to neighbors	malfunctioning	*A*_*1*_
	Intentionally discarding data (orders)	malicious	*A*_*2*_
Tampering with data (orders)	Not modifying data (orders)	normal	*A*_*3*_
	Not modifying data (orders), but network transmission error	malfunctioning	*A*_*4*_
	Intentionally modifying data (orders)	malicious	* A*_*5*_
Replaying or forging data (orders)	Not replaying or forging data (orders)	normal	*A*_*6*_
	Not replaying or forging data (orders), but network transmission error	malfunctioning	*A*_*7*_
	Intentionally replaying or forging data (orders)	malicious	*A*_*8*_

**Fig 3:**
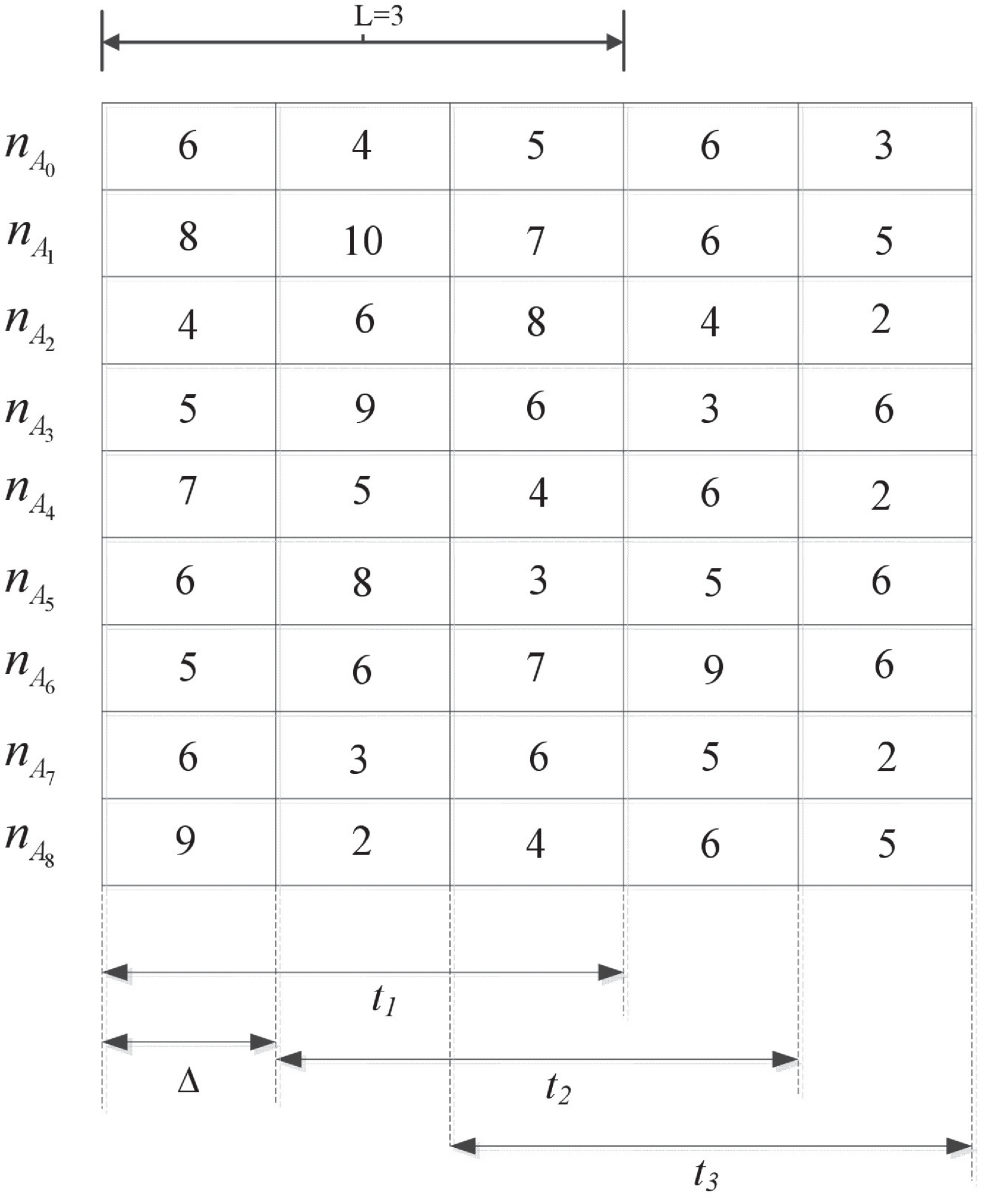
Example of time window mechanism in D-S scheme.

In order to adapt easily to multiple application scenarios, nine assumptions of interaction behavior are defined. The behavior of reader is divided into three levels: malicious reader, normal reader, malfunctioning reader. Let *R*_*j*_ denotes the neighbor node of reader *R*_*i*_. Let $T_{{}_{ji}}^{lo}(t_{k})$ denotes the local trust of *R*_*i*_ that is evaluated by its neighbor node *R*_*j*_ in time window *t*_*k*_. Here, we introduce time window mechanism, and the main objective of the timing window is to record recent records and forget previous records [25]. The time window in Fig 3 consists of three time units (L = 3).

**Fig 4:**
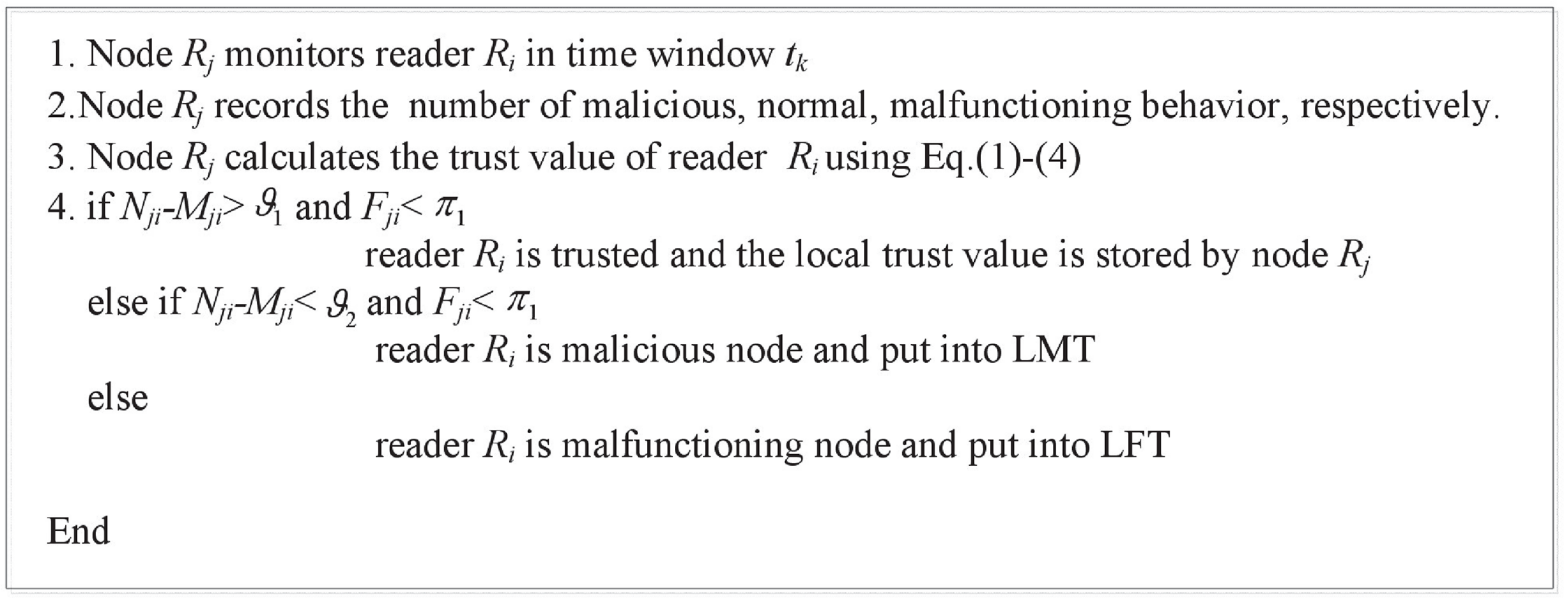
Algorithm of computing the local trust of reader.

In time window *t*_*k*_, neighbor node *R*_*j*_ records the number of interaction behavior of *R*_*i*_, and uses them to compute $T_{{}_{ji}}^{lo}(t_{k})$ as follows:

$$N_{ji}=\frac{n_{A_{0}}+n_{A_{3}}+n_{A_{6}}}{n_{all}}$$ (1)

$$M_{ji}=\frac{n_{A_{2}}+n_{A_{5}}+n_{A_{8}}}{n_{all}}$$ (2)

$$F_{ji}=\frac{n_{A_{1}}+n_{A_{4}}+n_{A_{7}}}{n_{all}}$$ (3)

$$T_{{}_{ji}}^{lo}(t_{k})=(N_{ji},M_{ji},F_{ji})$$ (4)

where:

$n_{A_{0}}....n_{A_{8}}$: the number of interaction behavior *A*_*0*_…*A*_*8*_;
*n*_*all*_: the total number of all interaction behavior;
*N*_*ji*_: the reader *R*_*i*_’ local trust value of normal behavior calculated by *R*_*j*_ in *t*_*k*_;
*M*_*ji*_: the reader *R*_*i*_’ local trust value of malicious behavior calculated by *R*_*j*_ in *t*_*k*_;
*F*_*ji*_: the reader *R*_*i*_’ local trust value of malfunctioning behavior calculated by *R*_*j*_ in *t*_*k*_;

The proposed algorithm of computing $T_{{}_{ji}}^{lo}(t_{k})$ is described in the following Fig 4.

As the example in Fig 3 shows, after each Δ period, the time window slides to the right, recording recent interaction behavior information and forgetting information recorded earlier. The time window in Fig 3 consists of three time units (L = 3), and $n_{A_{0}}....n_{A_{8}}$ are the number of interaction behavior *A*_*0*_…*A*_*8*_, respectively, of reader *R*_*i*_ observed by its neighbor node *R*_*j*_ in time window *t*_*k*_.

Every node maintains two tables: local malicious node table (LMT) and local malfunctioning node table (LFT). In Fig 4, *ϑ*_2_ < *N*_*ji*_−*M*_*ji*_ < *ϑ*_1_. *ϑ*_1_, *ϑ*_2_ and *π*_1_ is the trust threshold value. In order to prevent the malicious behavior, a high value is given to *ϑ*_1_ and *ϑ*_2_. *π*_1_ is used to evaluate the malfunctioning status of reader. In our simulation experiments, the value of *ϑ*_1_, *ϑ*_2_ and *π*_1_ are 0.7, 0.5, 0.3, respectively. After every Δ period, the time window slides to the right, recording recent information and forgetting information recorded earlier.

**Fig 5:**
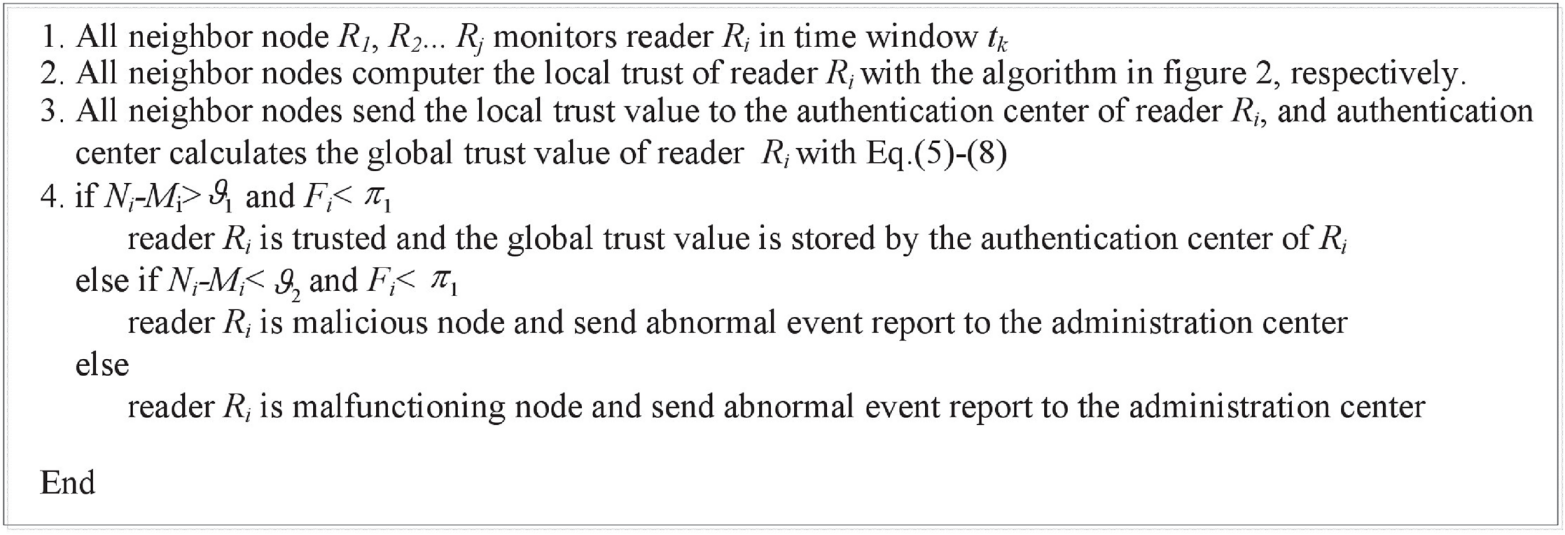
Algorithm of computing the global trust of reader.

The interaction events of a RFID reader can be observed by other neighbor nodes except neighbor node *R*_*j*_. We can get a global trust value of RFID reader by efficiently integrating the local trust opinions calculated by all neighbor nodes in time window *t*_*k*_. However, the local trust opinions of neighbors have strong subjectivity and uncertainty. Evidence theory proposed by Dempster and Shafer can briefly express the important conceptions, such as ‘uncertainty’ or ‘not-knowing’. Based on the Dempster knowledge rule in section 2.2, the global trust value of reader *R*_*i*_ is eventually obtained as follows:

$$N_{i}=N_{1i}⊕N_{2i}⊕N_{3i}....⊕N_{ji}$$ (5)

$$M_{i}=M_{1i}⊕M_{2i}⊕M_{3i}....⊕M_{ji}$$ (6)

$$F_{i}=F_{1i}⊕F_{2i}⊕F_{3i}....⊕F_{ji}$$ (7)

$$T_{{}_{i}}^{gl}(t_{k})=(N_{i},M_{i},F_{i})$$ (8)

where:

*N*_1*i*_….*N*_j*i*_: the reader *R*_*i*_’ local trust value of normal behavior calculated by neighbor node *R*_1_…..*R*_*j*_ in *t*_*k*_, respectively;
*M*_1*i*_….*M*_j*i*_: the reader *R*_*i*_’ local trust value of malicious behavior calculated by neighbor node *R*_1_…..*R*_*j*_ in *t*_*k*_, respectively;
*F*_1*i*_….*F*_j*i*_: the reader *R*_*i*_’ local trust value of malfunctioning behavior calculated by neighbor node *R*_1_…..*R*_*j*_ in *t*_*k*_, respectively;
*N*_*i*_: the reader *R*_*i*_’ global trust value of normal behavior in *t*_*k*_;
*M*_*i*_: the reader *R*_*i*_’ global trust value of malicious behavior in *t*_*k*_;
*F*_*i*_: the reader *R*_*i*_’ global trust value of malfunctioning behavior in *t*_*k*_;

The proposed algorithm of computing $T_{{}_{i}}^{gl}(t_{k})$ is described in the following Fig 5.

The global trust of reader *R*_*i*_ is calculated by its authentication center. In addition, the global trust value of reader *R*_*i*_ is stored in its authentication center.

In the end, the trust computing process of reader *R*_*i*_ based on D-S scheme is summarized as four steps: 1) The interaction event of reader *R*_*i*_ is detected by its neighbors; 2) The neighbor nodes of reader *R*_*i*_ calculate the local trust of *R*_*i*_ by using a time window mechanism and send the local trust value to the authentication center; 3) The authentication center of reader *R*_*i*_ calculates the global trust of *R*_*i*_ by synthesizing these local trust opinions based on the Dempster knowledge rule; 4) If the reader *R*_*i*_ is a malicious or malfunctioning node, the authentication center sends the abnormal event report to administration center.

### Trust evaluation of reader based on verification of interaction proof

The pre-condition to use D-S based trust evaluation scheme is that the interaction events can be monitored by neighbor nodes. However, the events sometimes may not be monitored by neighbors due to the limited communication range in RFID systems. In addition, the sparse distributed readers also lead to the low monitoring efficiency. Therefore, we propose another trust evaluation method of reader based on verification of interaction proof (VIP scheme) in the section. We assume the following scenario:

*R*_*i*_ and *R*_*j*_ are denoted as the readers. Let *C*_*A*_ and *C*_*B*_ to denote the authentication center of *R*_*i*_ and *R*_*j*_. *T*_*i*_ is denoted as a tag and its authentication center is *C*_*B*_. At time *t*, a reader *R*_*i*_ wants to interact with the tag *T*_*i*_.

The process of pre-authorizing is described in the following.

1. Reader *R*_*i*_ finds Tag *T*_*i*_, and sends the interaction request to *T*_*i*_, then *T*_*i*_ responds the request and sends the information about its number, name of its home domain, etc., to the *R*_*i*_.
2. After *R*_*i*_ receives the response information, it sends the authorization request to the authentication center of *T*_*i*_. The authentication center of *T*_*i*_ makes the interaction decision based on the trust of *R*_*i*_.
3. If the authorization is approved, the authentication center of *T*_*i*_ sends the authorization certificate to *R*_*i*_. Then, Reader *R*_*i*_ shows the authorization certificate to *T*_*i*_ and finishes the interaction at time *t*. Finally, *T*_*i*_ saves the interaction feedback record (*R*_*i*_,*t*,*S*_*i*_). *S*_*i*_ expresses feedback score. Tag *T*_*i*_ rates 1 if it is satisfied with the interaction and 0 otherwise.
4. At next time *t*', tag *T*_*i*_ interacts with Reader *R*_*j*_. *T*_*i*_ adds the interaction feedback record (*R*_*i*_,*t*,*S*_*i*_) to the data packet *D*, and delete the record in its own memory.
5. Then *D* is changed as *M*, where *M* = (*cer*_*t*'_,*rn*_*t*'_,*seq*,*R*_*i*_,*t*,*S*_*i*_,*h*) and *h* = *hash*(*cer*_*t*'_,*rn*_*t*'_,*seq*,*R*_*i*_,*t*,*S*_*i*_). *h* is hash function which ensures the integrity of *M*. *cer*_*t*'_ is the certificate of *T*_*i*_. *rn*_*t*'_ is random number of *T*_*i*_. *seq* is sequence number of *D*. *T*_*i*_ forwards *M* to *R*_*j*_. *R*_*j*_ adds $\left(cer_{R_{j}},rn_{R_{j}},h'\right)$ to *M*. *M* is changed as *M*'. $M'=(cer_{t'},rn_{t'},seq,R_{i},t,S_{i},cer_{R_{j}},rn_{R_{j}},h')$ and $h'=hash(cer_{R_{j}},rn_{R_{j}},h)$. $cer_{R_{j}}$ and $rn_{R_{j}}$ is the certificate and sequence number of *R*_*j*_, respectively. *M*' contains the sign of *R*_*j*_.
6. *R*_*j*_ forwards *M*' to an intermediate reader, which will check *h* and *h*'. If the checking fails, the intermediate reader will refuse to forward *M*', otherwise forwards *M*' to the authentication center *C*_*B*_ of reader *R*_*j*_.
7. After the authentication center *C*_*B*_ receives *M*', it will check whether there is an abnormal event of misusing the authorization or not at time *t* based on the feedback score. If the feedback score is 0, the authentication center *C*_*B*_ of reader *R*_*j*_ will send the abnormal event report to authentication center *C*_*A*_ of reader *R*_*i*_ and administration center, respectively.

**Fig 6:**
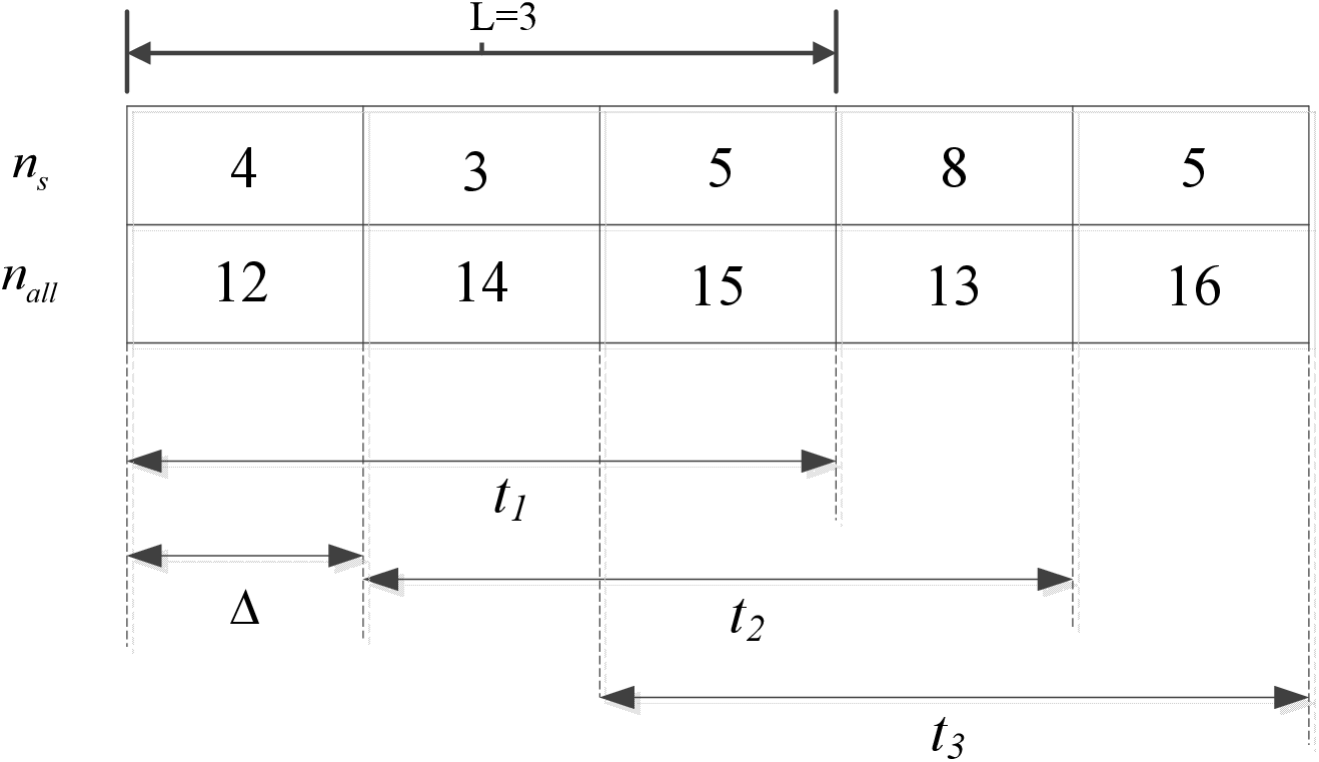
Ample of time window mechanism in VIP scheme.

Here, we also introduce the time window mechanism. Fig 6 shows the example of time window mechanism in VIP scheme.

In time window *t*_*k*_, the authentication center of reader *R*_*j*_ records the number of interaction behavior of *R*_*i*_, and uses them to compute the global trust value $T_{{}_{i}}^{gl}(t_{k})$ of reader *R*_*i*_ as follows:

$$T_{{}_{i}}^{gl}(t_{k})=1\text{-}\frac{n_{s}}{n_{all}}$$ (9)

where:

*n*_*s*_: the interaction number of score being 0 of reader *R*_*i*_ in time window *t*_*k*_;
*n*_*all*_: the all interaction number of reader *R*_*i*_ in time window *t*_*k*_;
$T_{{}_{i}}^{gl}(t_{k})$: the global trust value of reader *R*_*i*_.

The time window in Fig 6 consists of three time units (L = 3), and *n*_*s*_ and *n*_*all*_ are the interaction number of score being 0 and the all interaction number, respectively, of reader *R*_*i*_ in time window *t*_*k*_.

**Fig 7:**
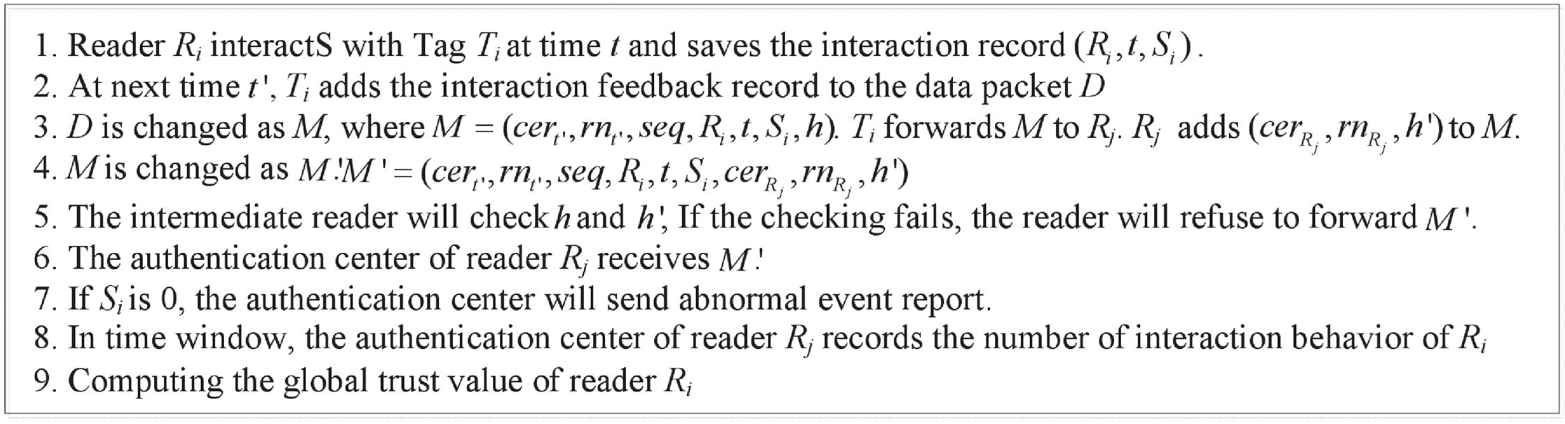
The realization process of VIP scheme.

Fig 7 expresses the details of VIP scheme. The proposed method can track the authorization use of reader by checking the interaction proof. The method avoids the impact of reader distribution and limited communication distance between readers and tags.

The trust computing process of VIP scheme is summarized as four steps: 1) The authentication center pre-authorizes reader *R*_*i*_ to interact with the tag *T*_*i*_; 2) The interaction feedback record at time *t* is saved in the tags; 3) At the time of next interaction, the tag *T*_*i*_ interacts with the reader *R*_*j*_.The interaction feedback record at time *t* is added into the data packet and transmitted to the authentication center of reader *R*_*j*_; 4) If the feedback score is 0, the authentication center of *R*_*j*_ will send the abnormal event report to authentication center of *R*_*i*_ and administration center, respectively.

Main advantages of the proposed method based on verification of interaction proof are:

1. The authentication center tracks the authorization use of a reader by checking the interaction feedback record.
2. The tag saves the interaction feedback record at time *t*. At the next time *t*', the interaction feedback record at time *t* is added into the data packet, and then tag deletes the record in its own memory. Only saving the recent interaction feedback record is suitable for limited built-in memory tag.
3. Intermediate readers will verify the integrity of data packet by checking *h* and *h*'. As a result, the proposed method guarantees the route security during the process of transmitting the data packet.
4. The proposed method can effectively prevent the tampering, replaying or forging attacks by checking *h*, adding random number and time stamp in the data packet.

### Trust management of authentication centers

The number of authentication centers is few, and their status is stable in a multi-domain RFID system. Therefore, a centralized trust evaluation scheme is proposed to evaluate the trustworthiness of authentication centers. An administration center is in charge of managing the trust of authentication center based on the abnormal event reports of readers of its own domain.

**Fig 8:**
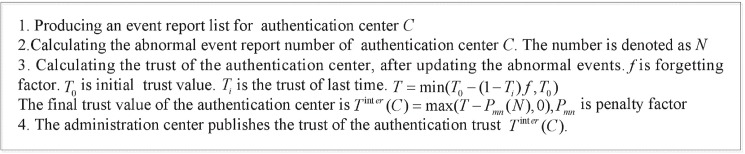
Computing the trust of authentication center.

The authentication center needs to collect the abnormal events of readers of its own domain periodically, and sends the abnormal event reports to administration center. The abnormal events can be found based on D-S scheme or VIP scheme. The administration center receives the abnormal event reports and computes the trust of authentication center, as shown in Fig 8.

Let *A* and *B* denote two different domains. *C*_*A*_ and *C*_*B*_ denote their authentication center, respectively. A tag *T*_*i*_ belongs to the domain *A*. Before a reader *R*_*i*_ interacts with a tag *T*_*i*_, *R*_*i*_ need to be authorized by the authentication center *C*_*A*_ of tag *T*_*i*_. *R*_*i*_ sends the authorization request to *C*_*A*_. If *R*_*i*_ and *T*_*i*_ is in the same domain *A*, *C*_*A*_ computes the trust of *R*_*i*_ as follows:

$$T_{{}_{i}}^{\mathrm{int}ra}(R_{i})=T_{{}_{i}}^{gl}(t_{k})$$ (10)

where $T_{{}_{i}}^{\mathrm{int}ra}(R_{i})$ is intra-domain trust, which can be obtained with Eq(9) or Eq(8).

If *R*_*i*_ and *T*_*k*_ isn’t in the same domain *A*. *R*_*i*_ belongs to the domain *B*. *C*_*A*_ computes the trust of *R*_*i*_ as follows:

$$T_{i}^{cross}(R_{i})=\beta T_{{}_{i}}^{\mathrm{int}ra}(R_{i})+(1-\beta )T_{{}_{B}}^{\mathrm{int}er}(C_{B})$$ (11)

where $T_{i}^{cross}(R_{i})$ is cross-domain trust. $T_{{}_{B}}^{\mathrm{int}er}(C_{B})$ is inter-domain trust, which is the trust of authentication center *C*_*B*_ of *R*_*i*_. $T_{{}_{B}}^{\mathrm{int}er}(C_{B})$ is computed by the administration center, as shown in Fig 8. *β* is weighting factor.

If reader *R*_*i*_ is malicious node or malfunctioning node, the authorization is refused, otherwise approved. When an abnormal event of *R*_*i*_ is found, the authentication center *C*_*A*_ will consider the behavior status of *R*_*i*_ as malicious reader or malfunctioning node, and send the abnormal event report to administration center. Then, the trust of authentication center of *C*_*B*_ is changed by the administration center.

## Experimental study

**Fig 9:**
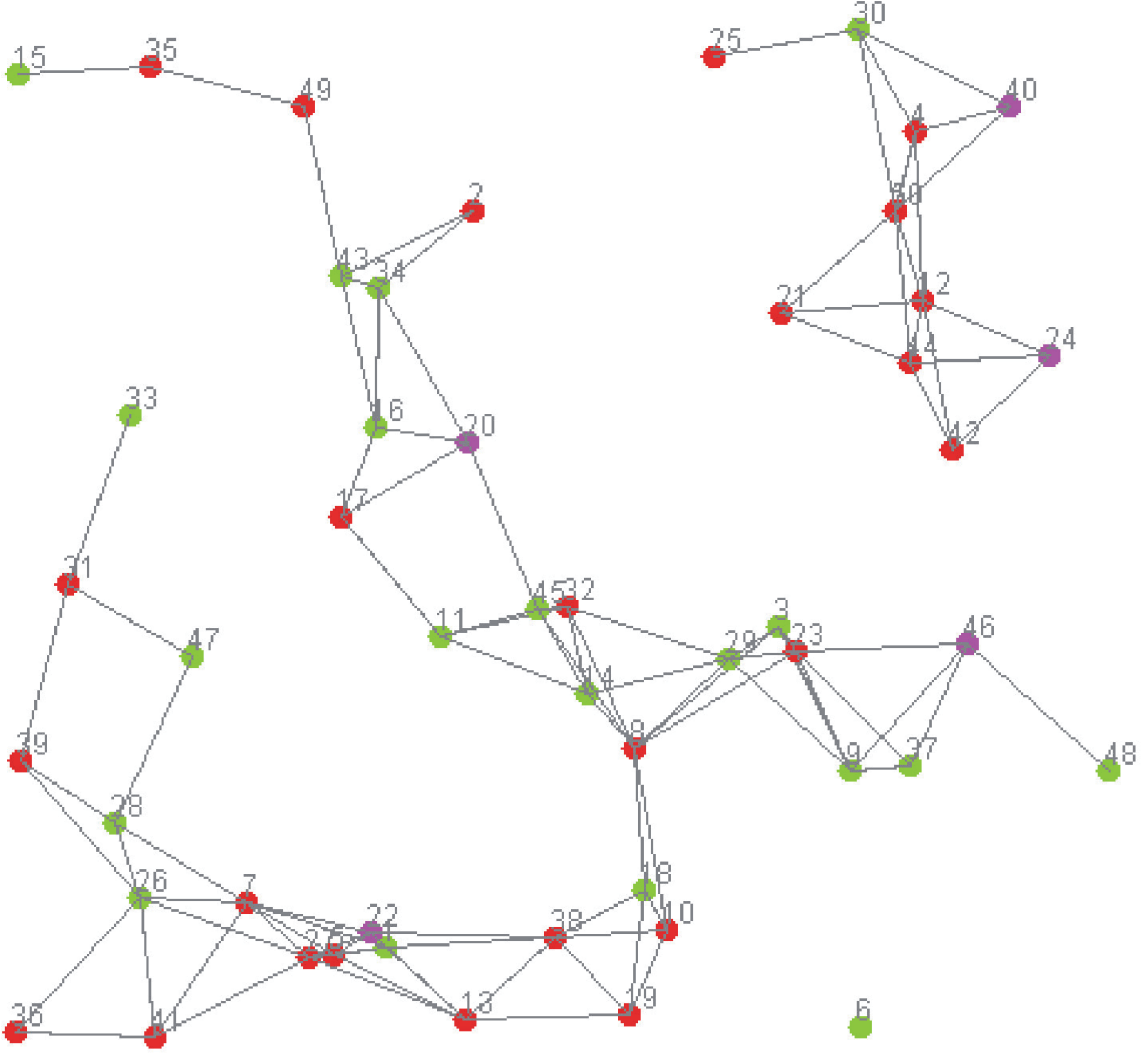
Network topology in the simulation experiments.

In this section, in order to evaluate the effectiveness of the proposed trust management, a series of test scenarios are developed. Experiments were run using the ns3 simulator [26] on which the creation of trust patterns, behaviors and interactions model was easier than with other network simulators. Fig 9 shows the network topology, where red, green and pink points express RFID readers, RFID tags and authentication centers respectively.

We assume that 100 readers are distributed at the area of three domains (*C*_*A*_, *C*_*B*_ and *C*_*D*_) whose size is 1500m x 1000m^2^. Each reader is located at a random position. Communication range of a reader and a tag is 200m and 70m. Here, we simulate active tags which have a wide transmission range of more than 70m [27]. The total simulation time is 260s. Firstly, trust evaluation accuracy is examined by comparing our schemes with other scheme [28]. In addition, we also study the effect of mobility and communication range of tag on detection rate of malicious event. Table 2 expresses the default simulations parameters.

**Table 2:** Default simulations parameters.

Number of readers	100
Number of tags	20
Communicating Range of a reader (m)	200
Communicating Range of a tag (m)	70
Simulation time (s)	260
Trust threshold *ϑ*_1_, *ϑ*_2_ and *π*_1_	0.7, 0.5, 0.5
Simulation Area (m^2^)	1500m x 1000m^2^
Communication Protocol	802.11
Not replaying or forging data (orders), but network transmission error	malfunctioning
Intentionally replaying or forging data (orders)	malicious
Maximum Speed (m/s)	70
Number of Malicious nodes	0%-50% of all nodes
Type of malicious event	Discarding data packet

### Accuracy of trust evaluation

Trust evaluation accuracy plays an important role of evaluation the performance of the trust scheme. In the section, we examine trust evaluation accuracy of D-S scheme and VIP scheme, and make comparisons with Bayes-based scheme [28].

**Fig 10:**
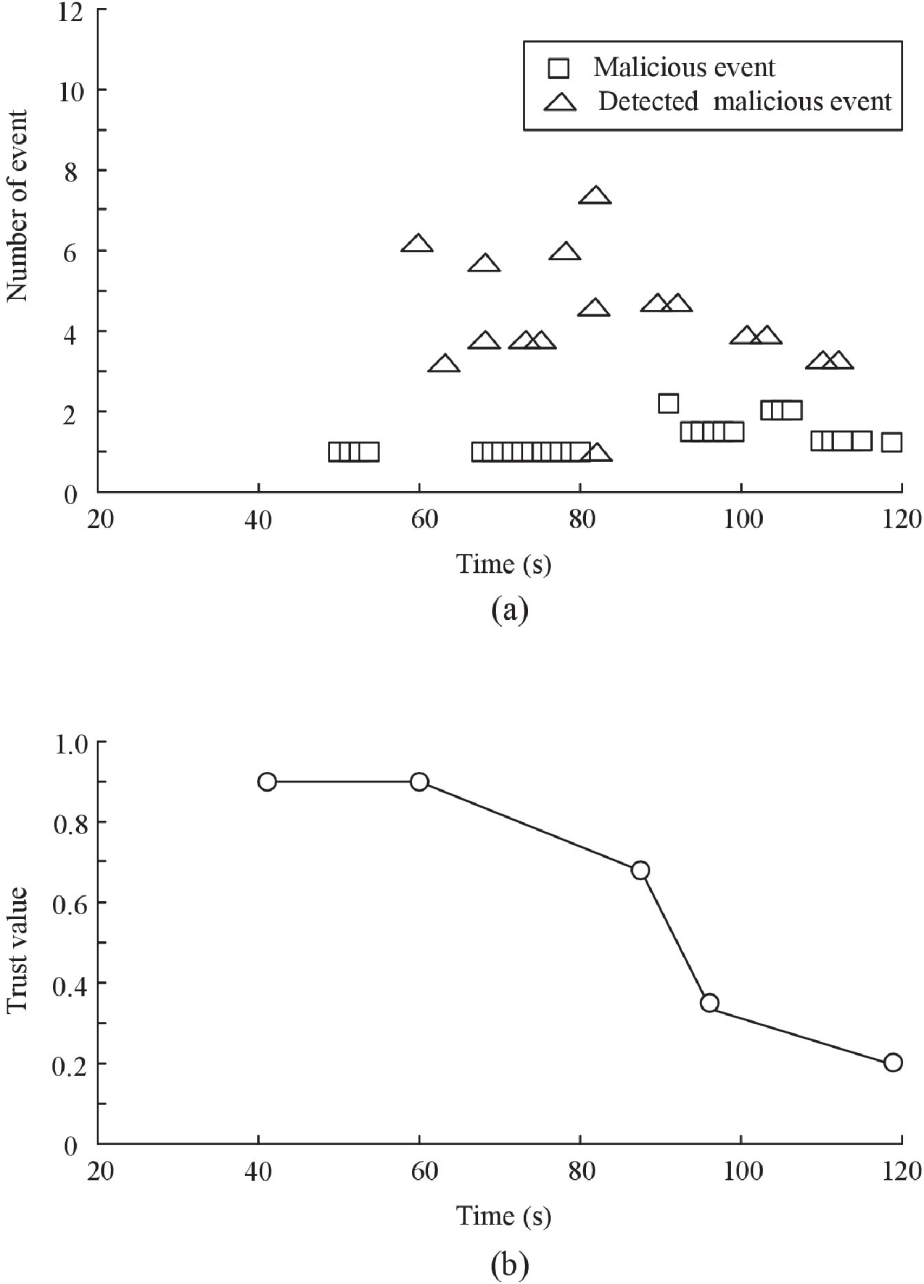
The convergence speed of D-S scheme. (a) Event result (Speed = 40m/s) (b) Trust result (Speed = 40m/s).

In the first group of experiment, 100 readers are distributed in the area of three domains (*C*_*A*_, *C*_*B*_ and *C*_*D*_) whose size is 900m x 600m^2^. Other parameters are default parameters. We vary fraction (*P*_*M*_) of malicious readers who discards data packet from as low as 10% to as high as 50%. A reader selected to be in this “malicious” population is benign initially, but turns malicious after a period of time t∈[0, 120s] randomly generated is elapsed. The initial trust value of authentication center *C*_*A*_ is 0.9. In the experiment, D-S scheme is used to evaluate the trust of *C*_*A*_. In our trust management framework, the trustworthiness of authentication center is evaluated by administration center. Based on trust evaluation algorithm in the system model section, the trust of authentication center is evaluated by collecting the abnormal event reports. The trust evaluation results are shown in Fig 10.

We can see that there are four malicious events at 50s, which are found by neighbor nodes at 60s, and six malicious event reports are sent to administration center. A malicious event may be detected by multi-neighbors, so there are multi-reports. Once administration center receives the malicious event reports, the trust value of *C*_*A*_ is immediately updated. As the report number of malicious events increases, the trust value of *C*_*A*_ drops quickly. We see that after the behavior status changes, our trust scheme quickly converges towards the new trust value. The reason is that using more malicious event reports helps trust convergence more quickly. Therefore our scheme can deal with large scale RFID applications.

**Fig 11:**
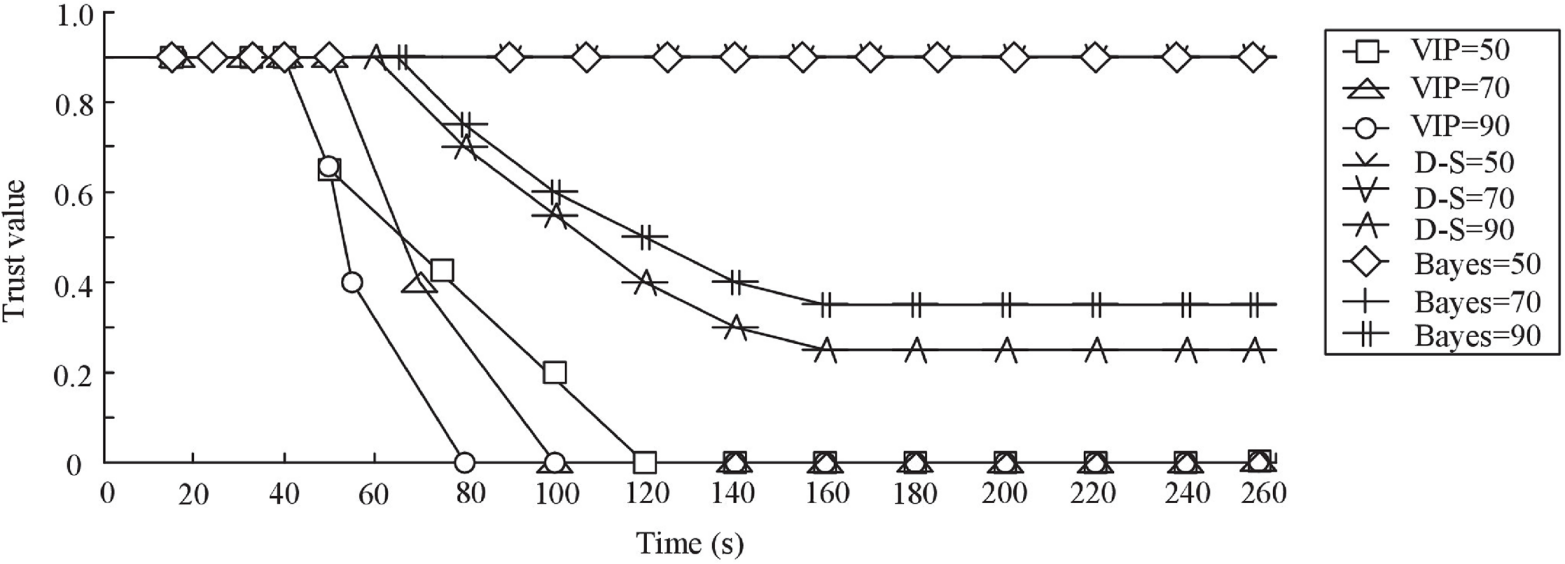
Comparation of convergence speed.

**Fig 12:**
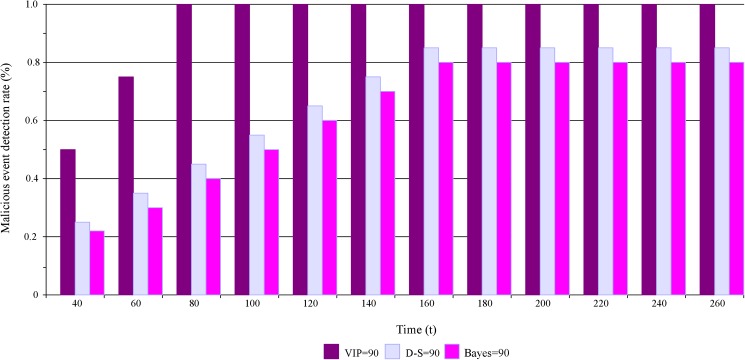
Comparation of malicious event detection rate.

In the second group of experiment, we compare our schemes with Bayes-based scheme. The trust of C_*A*_ is respectively evaluated three times by VIP scheme, D-S scheme and Bayes-based scheme. The number of readers is respectively 50, 70 and 90 every time. The fraction (*P*_*M*_) of malicious readers is 20%. A reader selected to be in this “malicious” population turns malicious after a period of time t∈[0, 120s]. Other parameters are default parameters. The results are shown in Fig 11 and Fig 12.

We can see that the trust value of C_*A*_ is changeless in the first and second time experiment of D-S scheme. But the trust value decreases in the third time experiment of D-S scheme. The reason is that the sparse distributed readers lead to the low malicious event detection rate. Because the number of readers is less than 90, the malicious events aren’t be detected by neighbor nodes. The trust evaluation results of D-S scheme and VIP scheme are similar. VIP scheme outperforms all other mechanisms, which detects earlier the node misbehavior and decreases the trust level of C_*A*_. Even if the number of readers is 50, the malicious events can also be detected by VIP scheme. D-S scheme and Bayes-based scheme adjust the trust value of C_*A*_ based on observing the communication behavior of readers. But, the behaviors sometimes may not be detected due to the limited communication range in RFID systems.

Fig 12 shows the results of malicious event detection rate. In the figure, the number of readers is 90. From Fig 12, we can see that as the time increases, the malicious behavior detection rate also rises. When time = 80s, the detection rate of VIP scheme reaches to the best value. When time = 160s, the detection rate of D-S and Bayes-based scheme reaches to the best value. The detection rate of malicious events in Bayes-based scheme is the lowest.

### Effect of mobility of tag

**Fig 13:**
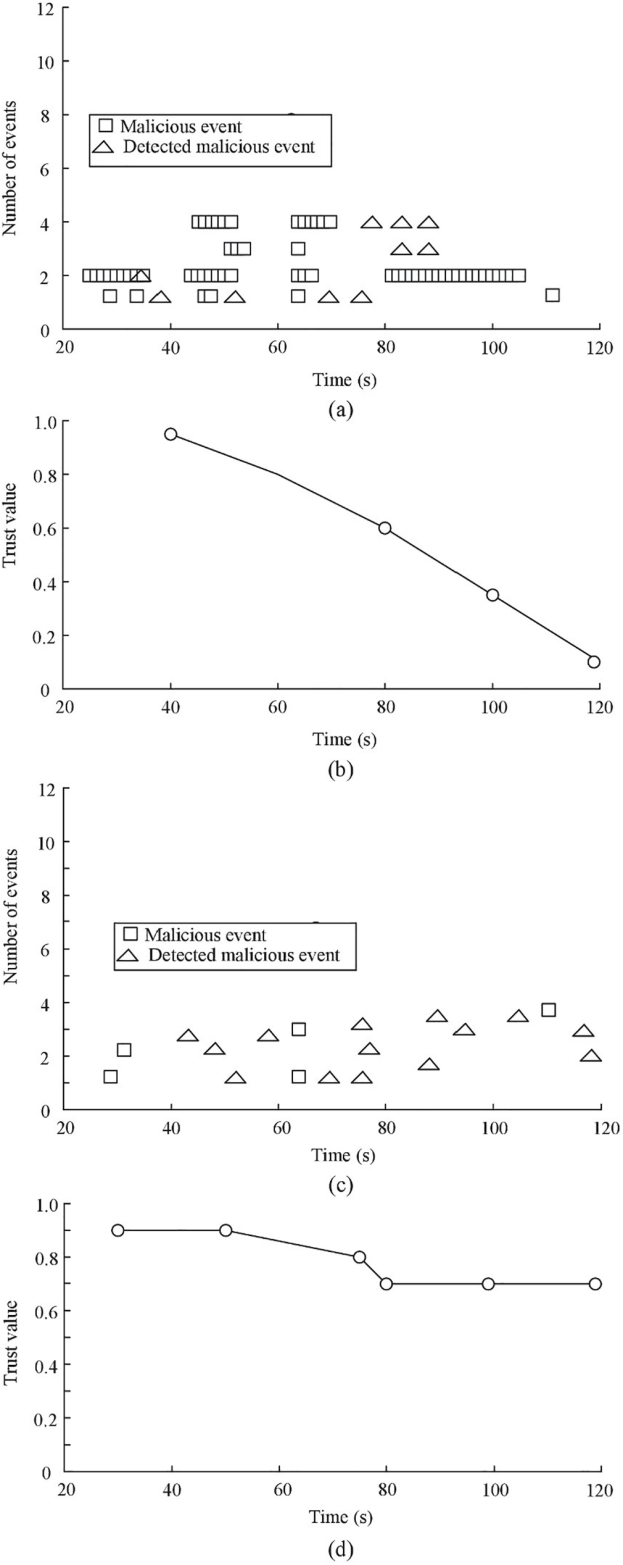
Effect of mobility of tag on detection rate of malicious event. (a) Event result (Speed = 15m/s) (b) Trust result (Speed = 15m/s) (c) Event result (Speed = 70m/s) (d) Trust result (Speed = 70m/s).

A tag is typically attached to an object that may roam to other administrative domains. The mobility of tag plays an important role when designing trust management mechanisms and protocols. Since the tag moves from one domain to another domain, the network topology also keeps continuously changing. These changes will have effect on detecting the malicious events. In the section we evaluate the effect of mobility of tag on detecting malicious event. Our experiments are divided into two groups. In the process of experiment, we use D-S scheme to evaluate the trustworthiness of reader. The fraction (*P*_*M*_) of malicious readers is 20%. The communication range of tag is 70m. In the two groups of experiments, the tags are moving continuously at 15m/sec and 70m/sec, respectively. Other parameters are default parameters. Fig 13(A)–13(D) shows the simulation results.

In the Fig 13, the square mark, triangle mark and circle mark respectively indicates the malicious event, detected malicious event and the trust value of *C*_*A*_. From Fig 13, one can see that as the moving speed of tags increases, the occurrence rate of malicious events visibly decreases, but the detection rate of malicious events becomes higher. Faster moving of tags leads to the shorter interaction time with readers. Thus, the average number of malicious events decreases. One can see that the average number of malicious events is respectively 120 and 40 in Fig 13(A) and Fig 13(C).

### Effect of communication range of tag

**Fig 14:**
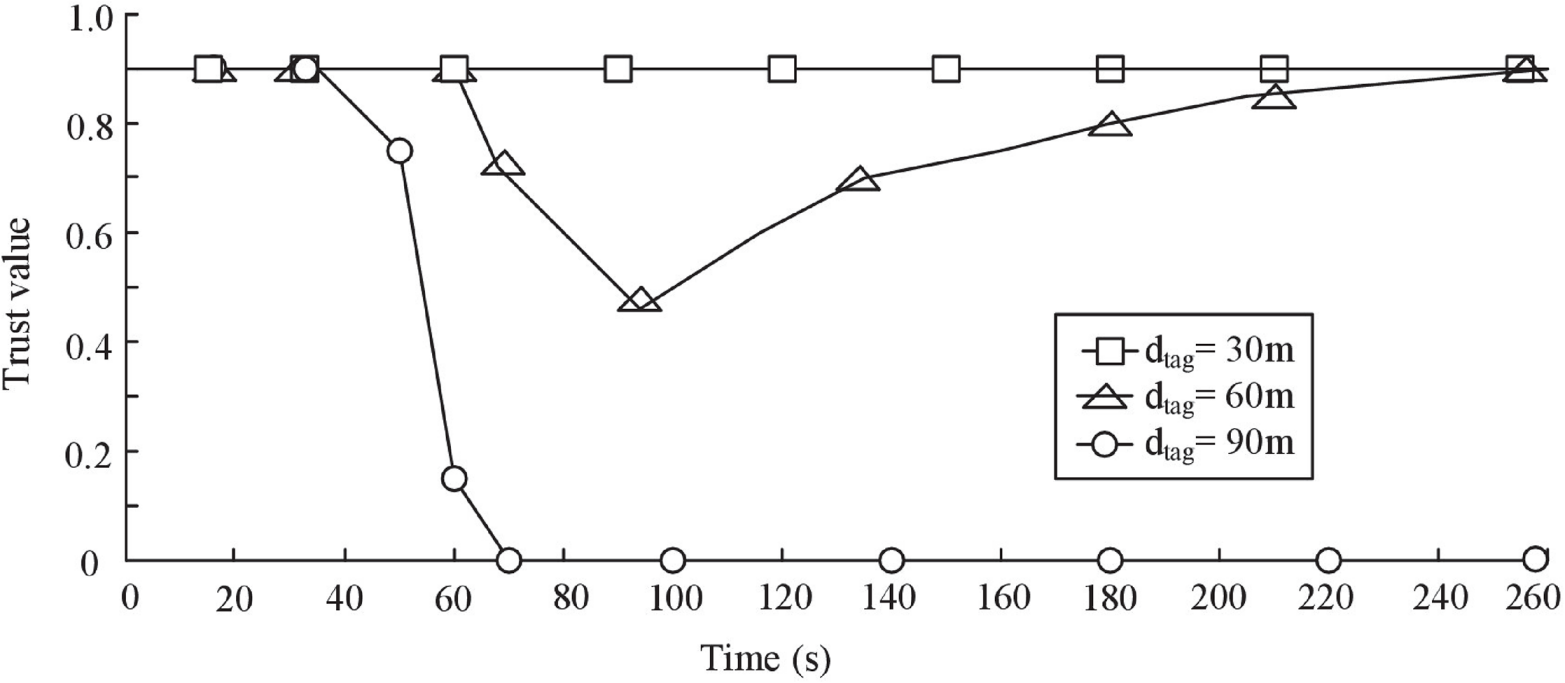
Effect of communication range of tag on detection rate of malicious event.

In the section, we evaluate the effect of communication range of tag on detection rate of malicious event. We assume that the communication range of tag is *d*_*tag*_. The experiment is simulated three times. Communication range of tag is set to 30m, 60m and 90m respectively. The fraction (*P*_*M*_) of malicious readers is varied from 10% to as 50%. We use D-S scheme to evaluate the trustworthiness of reader. Other parameters are default parameters. The Fig 14 shows the trust evaluation result of the authentication center *C*_*A*_.

We can see that the trust value of *C*_*A*_ hasn’t any changes in the first experiment (*d*_*tag*_ = 30m). As the malicious events increase in the second experiment (*d*_*tag*_ = 60m), the trust value of *C*_*A*_ starts to decrease. After a while, no new malicious event is detected, and then the trust value of *C*_*A*_ gradually increases. In the third experiment (*d*_*tag*_ = 90m), the trust value of *C*_*A*_ quickly drops to the lowest value and remain steady.

As shown in Fig 14, the trust evaluation results of *C*_*A*_ are different in three experiments. The main reason is analyzed in the following:

The number of readers is *n*. The network area of readers is *S*. $\bar{N}$ is the average number of readers met by a tag.

$$\bar{N}=\frac{d_{{}_{tag}}^{2}\times \pi \times n}{S}$$ (12)

When the communication range is 30m, $\bar{N}=0.29$. Thus, the interaction is difficult to be detected by other readers in the first experiment. As a result, the performance of D-S scheme is far from satisfied, if the communication range of tag is too short.

## Conclusions and future

In the multi-domain RFID paradigm, a mobile tag will potentially interact with numerous readers from different management domains for a coalition, as well as leverage available (foreign) infrastructure for information access while on the move. However, trust establishment among entities from heterogeneous domains without past interaction or prior agreed policy, is a challenge. Based on the specific requirements in multi-domain RFID systems, this paper focuses on the critical trust management issues and proposes a multi-domain trust management model. The proposed trust management model provides a hierarchical trust management framework include a diversity of trust evaluation and establishment approaches. We refer to two layers of trust in the framework: RFID reader trust layer and authentication center trust layer. In RFID reader trust layer: We propose two kinds of scheme to evaluate the trust of readers: D-S evidence theory based scheme (D-S scheme) and verification of interaction proof based scheme (VIP scheme). In authentication center trust layer: An administration center is used to manage the trustworthiness of authentication centers in a centralized way. In the experiment section, we compare our schemes with Bayes-based scheme. The simulation results and analysis show that VIP scheme outperforms all other mechanisms, which detects earlier the node misbehavior. The detection rate of malicious events in Bayes-based scheme is the lowest. In addition, the performance of D-S scheme is far from satisfied, if the communication range of tag is too short. The malicious behaviors in D-S scheme and Bayes-based scheme sometimes may not be detected due to the limited communication range in RFID systems.

There are a few directions for our future work. In future work, the value of *ϑ*_1_, *ϑ*_2_ and *π*_1_ will be studied in the algorithm simulation. We plan to develop a full list of threats against the proposed hierarchical trust management framework and analyze the vulnerability of the system to these threats. Performance optimization of the trust management system is another focus of our future research work.

## Supporting information

S1 Dataset (ZIP)
